# The Neural Correlates of Facial Attractiveness in Resume Screening: Evidence from ERPs

**DOI:** 10.3390/bs15081130

**Published:** 2025-08-20

**Authors:** Bin Ling, Yuting Xia, Yihan Wang

**Affiliations:** School of Business, Hohai University, 8 West Focheng Road, Nanjing 211100, China; xiayuting1029@163.com (Y.X.); 221313040043@hhu.edu.cn (Y.W.)

**Keywords:** facial perception, attractiveness, employability, applicant evaluation, ERP

## Abstract

Facial attractiveness plays a significant role in job search evaluations, with recruiters often rating candidates with higher levels of attractiveness more favorably. This paper investigates how physical appearance and employability jointly influence applicant evaluations during resume screening. Using event-related potential (ERP) techniques, the study observes dynamic brain changes during the experiment. The findings reveal that: (1) Employability significantly enhances P200 amplitudes (reflecting early attentional allocation), while its effects on N170 and LPP components are contingent upon attractiveness levels; (2) These employability effects are selectively modulated by facial attractiveness: under high-attractiveness conditions, high employability potentiates both P200 and LPP responses (suggesting enhanced motivational engagement and emotional arousal); low employability leads to more negative N170 amplitudes (indicating early conflict detection to stereotype-incongruent cues). Conversely, no such effects emerge under low-attractiveness conditions, demonstrating that facial attractiveness gates the neural prioritization of qualification information. These results provide valuable insights into job search evaluations and highlight the neural mechanisms involved in facial perception and processing during resume screening.

## 1. Introduction

Facial attractiveness significantly influences social perceptions and interactions across various contexts, including hiring outcomes (H. Li et al., 2022). Defined as the extent to which a person’s face elicits positive emotional responses (Revers et al., 2023), it can encourage favorable evaluations in professional contexts. Research consistently shows that individuals with more attractive appearances are often evaluated more favorably during the recruitment process, enjoying greater access to employment opportunities (Brooks et al., 2014; J. Li et al., 2023)—a phenomenon frequently explained by the halo effect. This effect posits that physical attractiveness triggers a cognitive heuristic where positive perceptions of one trait (e.g., appearance) spill over to unrelated traits, leading recruiters to assume attractive candidates possess positive traits such as competence and trustworthiness (Theodoridou et al., 2009).

Signaling theory offers another framework for understanding how physical appearance influences recruitment evaluation. Signaling theory suggests that job candidates convey unobservable qualities through observable cues (Connelly et al., 2011; Spence, 1973). Especially in situations of information asymmetry—such as initial recruitment screenings—attractive appearance may be unconsciously interpreted as a “signal” of underlying qualities. From an evolutionary and social perspective, attractiveness is subtly associated with traits like health (Langlois et al., 2000), social adaptability (Rule & Ambady, 2008), or resourcefulness (Zebrowitz & Montepare, 2008), leading perceivers to infer that attractive individuals possess the capacity to meet job demands, even when no direct evidence supports this link. Additionally, another research highlights the existence of looks discrimination in the labor market (Kukkonen et al., 2023), with less attractive individuals facing biases that limit their earning potential and career advancement. However, while these findings underscore the pervasive role of facial attractiveness, much of the existing research examines it in isolation, without considering its interaction with explicit qualifications like employability.

This gap is critical because applicant evaluations rely on both explicit factors, such as education and work experience, and implicit cues, such as physical appearance (Fraenkel, 2022). Explicit factors, like educational background, skills, and achievements, are clearly outlined in resumes, making them tangible and measurable indicators of a candidate’s qualifications (Guilbert et al., 2015). In contrast, implicit factors, such as facial features, are not explicitly stated but can be inferred through elements like professional headshots (Y. Li et al., 2021). Despite growing evidence that facial attractiveness influences hiring decisions, the mechanisms by which it interacts with employability, defined here as the combination of explicit factors that signal a candidate’s readiness and suitability for a job, remain poorly understood. Existing research has yet to address how these two dimensions—facial attractiveness and employability—jointly shape applicant evaluations, leaving a significant gap in understanding the biases that drive hiring decisions.

This interaction raises key questions about how candidates are evaluated. Peng et al., for example, introduces the concept of an “ugliness premium,” suggesting that less attractive individuals may be perceived as compensating for physical shortcomings with greater effort and competence (Peng et al., 2020). This implies that varying levels of facial attractiveness can significantly shape recruiters’ judgments regarding an applicant’s employability and job fit. Given that job seekers communicate their abilities through resumes, and employers interpret these signals to assess potential, understanding the combined impact of facial attractiveness and employability is essentially—particularly regarding how these factors jointly influence evaluations and what neural processes underpin such judgments.

To address this critical need, the present study focuses on two core questions:

RQ1: How do facial attractiveness and employability jointly influence recruiters’ evaluations of job applicants, such as hiring recommendations?

RQ2: What are the neural mechanisms underlying the interaction between facial attractiveness and employability during resume screening?

These questions are critical because they bridge the gap between behavioral observations of hiring biases and their subconscious neural origins. Since physical appearance is often subconsciously linked to competence (Han et al., 2023), exploring neural responses to facial attractiveness in resume screening can clarify how recruiters process visual cues alongside explicit qualifications, shedding light on how both facial appearance and employability jointly influence applicant evaluations. Such neural-level investigation not only fills a critical research gap but also highlights how cognitive and affective processes contribute to hiring inequities.

This study is innovative in its approach to bridging these gaps by integrating behavioral and neuroscientific methods. Using event-related potentials (ERPs), a highly sensitive tool for measuring real-time brain activity (T. Li et al., 2024), we aim to uncover the temporal dynamics of neural responses to facial attractiveness during resume screening. Electroencephalography (EEG), with its high temporal resolution, identifies the stages of visual information processing in the brain, providing crucial insights into neural responses to visual stimuli. Using ERP technology, researchers have identified early ERP components evoked by human faces, even at the initial stages of visual perception. For instance, components such as N1 and N170 are relatively stable indicators of face processing (Baker et al., 2023; McCrackin & Itier, 2018).

The N170 component (peaking 130–200 ms post-stimulus) represents the brain’s earliest specialized response to faces, serving as a robust marker of face-selective perception. This component is modulated both by top-down cognitive influences from the prefrontal cortex (Eimer, 2000; Wu et al., 2023) and by socially relevant facial characteristics, such as attractiveness (Cao et al., 2015; Schindler et al., 2023). Crucially, attractive faces elicit significantly enhanced N170 amplitudes compared to less attractive faces, suggesting they automatically capture attention due to their heightened perceptual salience in social contexts (Wiese et al., 2014). Following the N170, the P200 (150–250 ms) is implicated in the initial affective appraisal of stimuli. The P200 is thought to index early valence discrimination (e.g., approach-avoidance tendencies; Horat et al., 2018), bridging perceptual encoding and subsequent evaluative stages. Later-stage processing is captured by the LPP (300–700 ms), a sustained positive deflection linked to motivated attention and memory consolidation. The LPP originates from temporal-parietal networks (Polich, 2007) and is amplified by emotionally salient stimuli, including attractive faces (Marzi & Viggiano, 2010). This component reflects deeper socioaffective engagement—such as reward anticipation (Huerta-Chavez & Ramos-Loyo, 2024) or social motivation—and is critical for understanding how attractiveness biases long-term encoding and decision-making (e.g., hiring contexts). Overall, these findings demonstrate that the brain exhibits distinct temporal responses to faces with varying levels of attractiveness, with observable differences in both early and late ERP components.

To further investigate, we designed a 2 (facial attractiveness: high vs. low) × 2 (employability: high vs. low) within-subject factorial experiment to examine the interactive effects of facial attractiveness and perceived employability on applicant evaluation processes. This experimental design aims to disentangle the complex interplay between these variables and explore their implications for recruitment practices. The dependent variables measured include job candidate evaluations and ERP components.

By integrating neural data with behavioral outcomes, this research provides a novel perspective on how implicit biases, such as those driven by facial attractiveness, intersect with explicit qualifications during hiring decisions. Ultimately, this study contributes to the broader effort to understand and mitigate biases in recruitment. By elucidating the cognitive mechanisms underlying hiring decisions, it offers actionable insights to foster fairer and more equitable hiring practices.

## 2. Materials and Methods

### 2.1. Participants

A priori power analysis was conducted using G*Power (version 3.1) (Faul et al., 2007) to determine the required sample size for a repeated measures ANOVA with two within-subject factors, each having two levels. The analysis assumed a medium effect size (*f* = 0.25), an alpha level of *α* = 0.05, and a power of 1 − *β* = 0.80. The nonsphericity correction was set to ε = 0.67^1^, and a moderate correlation (*r* = 0.50) between repeated measures was assumed. Results indicated that a minimum of 31 participants would be required to detect a medium-sized effect. 

In the present research, thirty-six undergraduate students majoring in Human Resource Management participated in this study to earn course credit. All participants had prior corporate internship experience and were familiar with resume screening processes. Consistent with standard ERP research protocols, we recorded participants’ handedness (all right-handed) to account for potential neural lateralization effects. And they all had normal or corrected-to-normal vision. Before the experiment, each participant signed an informed consent form, detailing that the study posed no risks to their well-being. The Ethics Committee of the first author’s university approved the study protocol. Participation was voluntary, and participants were free to withdraw at any time, in which case their data would be excluded from analysis. We excluded four participants because of excessive artifacts contained in their data, resulting in thirty-two valid participants (13 males, 19 females; age range = 19–25 years, *M* = 21.36, *SD* = 1.19).

### 2.2. Stimuli

In order to manipulate facial attractiveness, we selected 32 neutral-expression facial images from the CAS-PEAL large-scale Chinese face image database (Gao et al., 2007). These images were uniformly processed to remove external features such as hair, ears, and neck, leaving only internal features, including the eyes, nose, mouth, and cheeks. All images were presented in greyscale on a white background, resized to 367 × 480 pixels, and shaped into ellipses of identical size.

To determine the average attractiveness level of the selected faces, we conducted a pre-survey. We distributed a questionnaire to 80 college students (35 males) aged 18–25 (*M* = 20.23, *SE* = 1.46), asking them to rate the attractiveness of the 32 face images (16 Chinese males and 16 Chinese females) on a scale from 1 to 7, where 1 represented the lowest attractiveness, 4 represented average attractiveness, and 7 represented the highest attractiveness ($M_{high\;facial\;attractiveness}$ = 4.78, $M_{low\;facial\;attractiveness}$ = 2.55, *p* = 0.000).

Previous research has demonstrated a positive correlation between internship experience and employability (Jackson et al., 2024). In this experiment, employability was manipulated based on the company where the job seeker had completed their internship. Specifically, “internship experience in a top 500 enterprises of China” was used to represent a high level of employability, while “internship experience in other (non-top 500) companies” was used to indicate a low level of employability. To ensure consistency, sixteen images (1080 × 720 pixels) were created, each featuring a uniform white background and black font. The company names were standardized to have the same word count, ensuring uniformity across conditions ($M_{high\;employability}$ = 4.54, $M_{low\;employability}$ = 1.77, *p* = 0.000). See Appendix A for the list of enterprises names. Prior to the main experiment, all participants completed a validated questionnaire using a 5-point Likert scale (1 = “completely unrepresentative” to 5 = “fully representative”) to evaluate how effectively internships at China’s Top 500 versus non-Top 500 enterprises signaled employability. Results showed significantly higher ratings for Top 500 enterprises (*M* = 4.54, *SD* = 0.41) compared to others (*M* = 1.77, *SD* = 0.62; *p* < 0.001).

### 2.3. Procedure

The experimental program was developed using E-Prime 3.0. The design included four conditions based on the combination of facial attractiveness and employability: (1) high facial attractiveness/high employability, (2) high facial attractiveness/low employability, (3) low facial attractiveness/high employability, and (4) low facial attractiveness/low employability. The experiment was structured into six blocks, each containing 32 trials, for a total of 192 trials.

**Figure 1:**
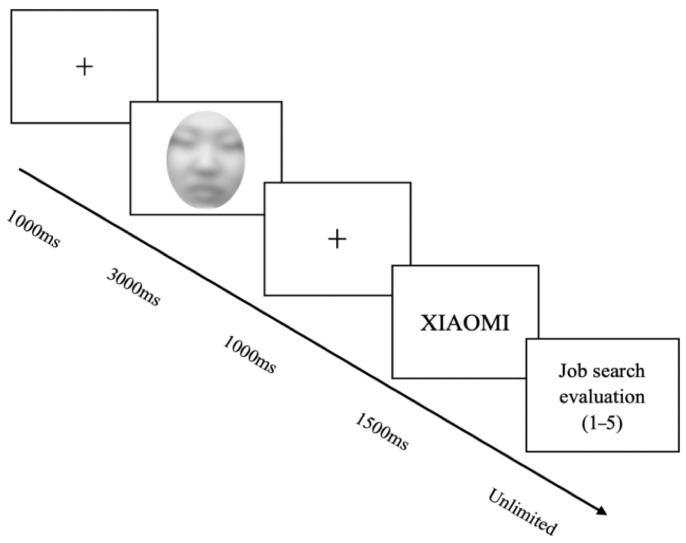
Experimental procedure.

At the start of the experiment, participants were informed that they would take on the role of a recruiter and would need to decide whether to offer a candidate an interview based on the candidate’s profile, which included a facial image and details of their internship experience. As shown in Figure 1, the formal experiment began when participants pressed any key to proceed. Each trial began with the presentation of a fixation cross (“+”) in the center of the screen for 1000 ms, followed by a face image in the same position for 3000 ms. A second fixation cross was then presented for 1000 ms, followed by an image depicting the candidate’s internship experience for 1500 ms. After viewing the images, participants made their applicant evaluation by rating the candidate on a five-point scale (1 = not willing at all, 5 = very willing) in response to the question: “To what extent would you recommend this candidate for the next round of interviews?”

Participants were instructed to minimize eye movements during the stimulus presentation to ensure accurate attention and response during the evaluation process. The experiment began with eight practice trials, after which participants proceeded to the formal experiment. A 1-min break was provided between each block. At the end of the experiment, participants were asked to complete a questionnaire regarding their personal information.

### 2.4. EEG Recording and Analysis

In this study, EEG data were collected using a Brain Products 64-channel system, with electrodes placed according to the International 10–20 System Extension. Electrode impedance was kept below 5 kΩ, and the recording bandwidth ranged from 0.1 to 40 Hz, with a sampling rate of 500 Hz per channel. The FCz electrode served as the reference, and the ground electrode was placed on the mid-forehead. Horizontal electrooculograms (HEOG) were recorded at the outer canthi of both eyes, and vertical electrooculograms (VEOG) were recorded above and below the left eye. Behavioral data were simultaneously recorded during the EEG acquisition process.

Applicant evaluation scores (RESP scores) were analyzed as the primary behavioral indicators. Due to unforeseen circumstances, five participants withdrew from the experiment, resulting in invalid data. Therefore, the data from the remaining 32 participants were analyzed using a two-way repeated-measures analysis of variance (ANOVA) in SPSS 26.0. The behavioral analysis examined the effects of facial attractiveness (high vs. low) and employability (high vs. low) on RESP scores.

The recorded EEG data were analyzed offline using Brain Vision Analyzer 2.2 software. The EEG data underwent the following six sequential processing steps: (1) filtering (band-pass filter 0.1–40 Hz, zero-phase delay), (2) correction of vertical and horizontal electrooculograms using Independent Component Analysis (ICA) in semi-automatic mode, (3) segmentation (−200 to 1000 ms), (4) baseline correction (0 to 200 ms), (5) artifact removal, and (6) superimposed averaging. The average number of superimpositions exceeded 32 for each experimental condition, resulting in four types of averaged files corresponding to the 2 (facial attractiveness: high vs. low) × 2 (employability: high vs. low) conditions: S3, S6, S9, and S12. Peak detection was also performed as part of the analysis.

For the analysis of ERP components, we focused on the mean wave amplitudes within specific time windows. The N170 component was observed from 120 to 190 ms, with electrode sites Fz, F1, and F3 in the prefrontal region. The P200 component was examined from 200 to 250 ms at the Fz electrode site. For the LPP component, the mean wave amplitude was observed from 650 to 850 ms, with electrode sites Cz, C3, and C4. A two-way repeated-measures ANOVA was conducted on the mean wave amplitudes of the N170, P200, and LPP components, with *p*-values corrected using the Greenhouse-Geisser method.

## 3. Results

### 3.1. Behavioral Results

Applicant evaluation scores were used as the dependent variable, and a 2 × 2 repeated measures ANOVA was conducted. As summarized in Table 1, the results revealed a significant main effect of facial attractiveness, *F*(1, 31) = 127.96, *p* = 0.000, ${\eta^{2}}_{p}=$ = 0.805. Participants rated candidates with high facial attractiveness (*M* = 3.74, *SE* = 0.05) more favorably than those with low facial attractiveness (*M* = 2.90, *SE* = 0.08). A significant main effect of employability was also found, *F*(1, 31) = 32.65, *p* = 0.000, ${\eta^{2}}_{p}=$ = 0.513, Candidates with high employability (*M* = 3.71, *SE* = 0.09) received higher evaluation scores compared to those with low employability (*M* = 2.93, *SE* = 0.09).

**Figure 2:**
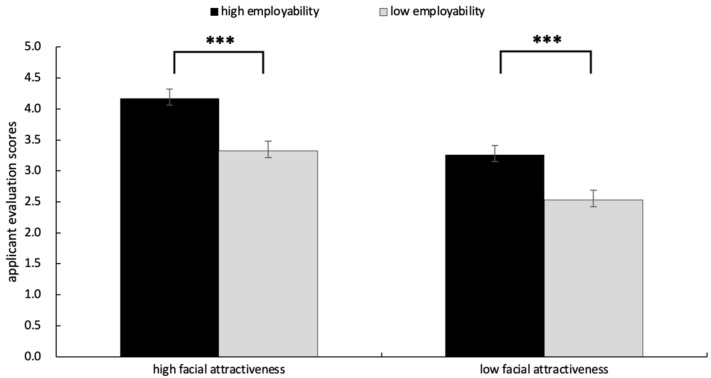
The results of applicant evaluation scores in each condition. Error bars represent standard errors (*** *p* < 0.001).

Importantly, the interaction between facial attractiveness and employability was significant, *F*(1, 31) = 4.93, *p* = 0.034, ${\eta^{2}}_{p}=$ 0.137, As illustrated in Figure 2, simple effects analysis revealed that under the high facial attractiveness condition, candidates with high employability received higher job scores than those with low employability, *F*(1, 31) = 12.78, *p* = 0.000, $M_{high\;employability}$ = 4.17, $M_{low\;employability}$ = 3.32. Under the low facial attractiveness condition, candidates with high employability received higher job scores than those with low employability, *F*(1, 31) = 7.32, *p* = 0.000, $M_{high\;employability}$ = 3.26, $M_{low\;employability}$ = 2.53.

### 3.2. ERP Results

#### 3.2.1. N170 Component (120–190 ms)

**Figure 3:**
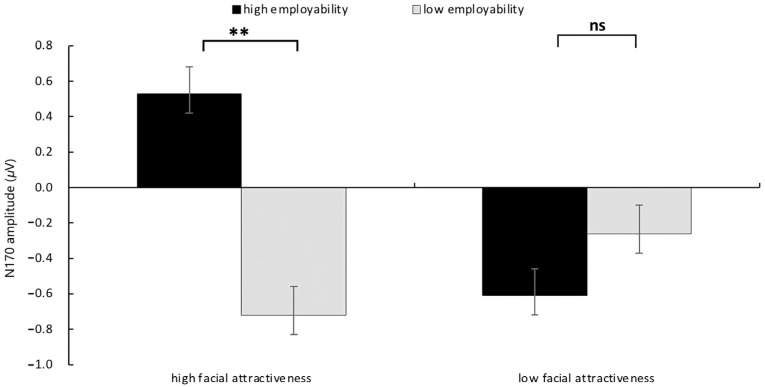
The results of N170 amplitude in each condition. Error bars represent standard errors (n.s.: nonsignificant, ** *p* < 0.01).

**Figure 4:**
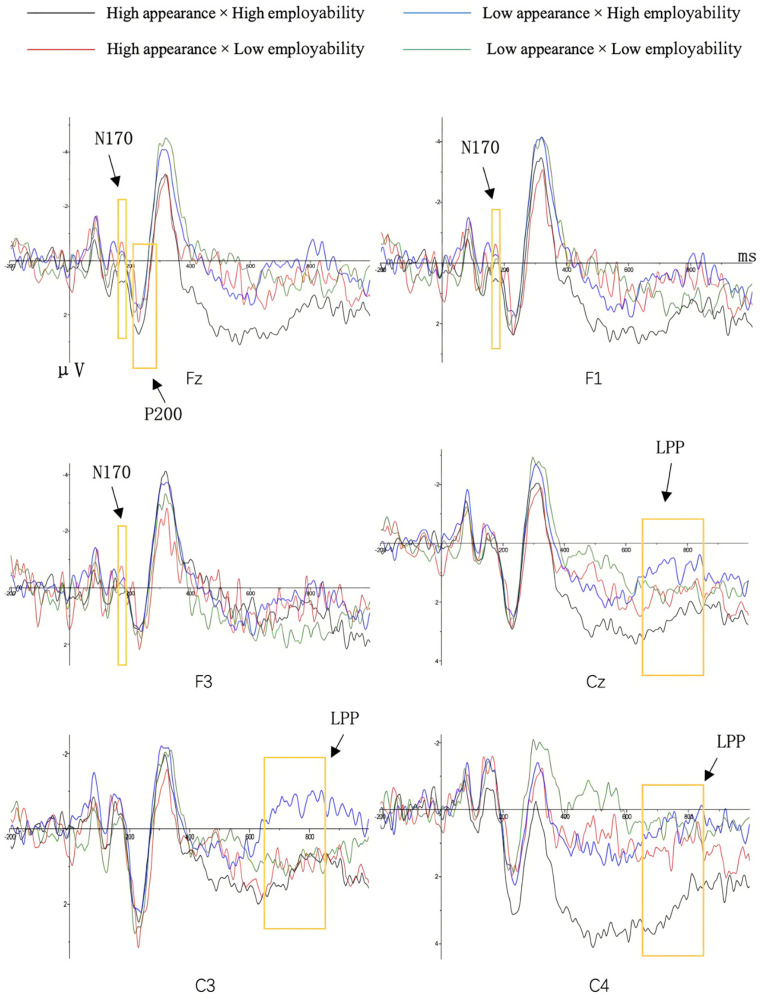
ERPs Results. The grand average waveforms at six representative electrodes in the midzone (FZ, F1, F3, CZ, C3, and C4) are presented. Positive voltage is plotted downward.

**Figure 5:**
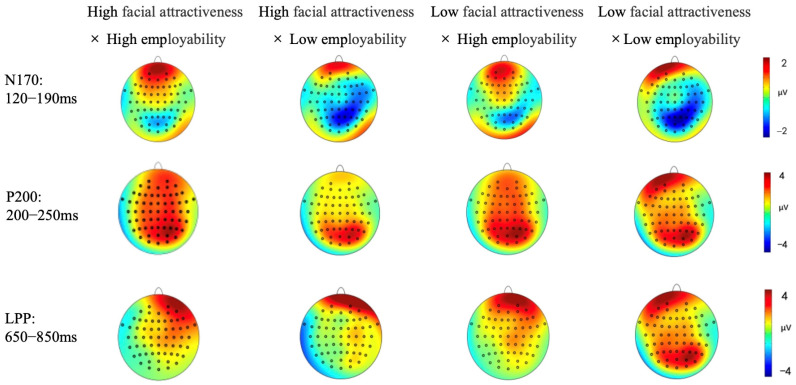
Scalp topographies.

The main effect of facial attractiveness and employability were both not observed, *F*(1, 31) = 0.63, *p* = 0.434, ${\eta^{2}}_{p}=$ 0.020, *F*(1, 31) = 2.04, *p* = 0.163, ${\eta^{2}}_{p}=$ 0.062. The interaction effect of facial attractiveness and employability on N170 amplitude was significant at the frontal electrodes (Fz, F1, F3), *F*(1, 31) = 4.66, *p* = 0.039, ${\eta^{2}}_{p}=$ 0.131. As illustrated in Figure 3, a simple effects analysis revealed that, under the high facial attractiveness condition, there is a significant N170 effect, *F*(1, 31) = 4.23, *p* = 0.003, ${\eta^{2}}_{p}=$ 0.120, the N170 amplitude induced by low employability (*M* = −0.72 μV, *SE* = 0.46) was significantly larger than that induced by high employability (*M* = 0.53 μV, *SE* = 0.36). However, under the low facial attractiveness condition, no significant difference was found between the high and low employability conditions (*p* = 0.54) (The ERP grand averaged waveforms and the topography are displayed in Figure 4 and Figure 5).

#### 3.2.2. P200 Component (200–250 ms)

**Figure 6:**
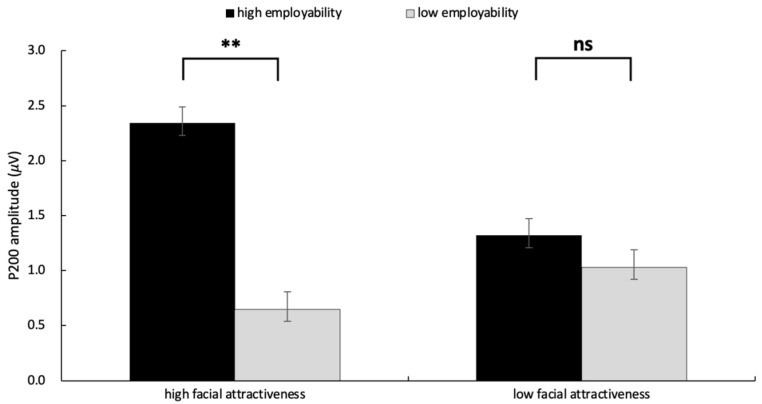
The results of P200 amplitude in each condition. Error bars represent standard errors (n.s.: nonsignificant, ** *p* < 0.01).

A significant main effect of employability was observed at the frontal electrode Fz, *F*(1, 31) = 5.45, *p* = 0.026, ${\eta^{2}}_{p}=$ 0.149, indicating that the P200 amplitude induced by high employability (*M* = 1.83 μV, *SE* = 0.49) was greater than that induced by low employability (*M* = 0.84 μV, *SE* = 0.56). The main effect of facial attractiveness was not observed, *F*(1, 31) = 0.38, *p* = 0.541, ${\eta^{2}}_{p}=$ 0.012. Additionally, the interaction between employability and facial attractiveness was significant, *F*(1, 31) = 4.31, *p* = 0.046, ${\eta^{2}}_{p}=$ 0.122. As illustrated in Figure 6, simple effect analysis revealed that, under the condition of high facial attractiveness, the P200 effect was observed, *F*(1, 31) = 5.45, *p* = 0.004, ${\eta^{2}}_{p}=$ 0.149, the P200 amplitude induced by high employability (*M* = 2.34 μV, *SE* = 0.65) was larger than that induced by low employability (*M* = 0.65 μV, *SE* = 0.65). However, under the condition of low facial attractiveness, there was no significant difference between the two levels of employability (*p* = 0.60) (The ERP grand averaged waveforms and the topography are displayed in Figure 4 and Figure 5).

#### 3.2.3. LPP Components (650–850 ms)

The main effect of facial attractiveness on the LPP component at the central electrode sites (Cz, C3, and C4) was significant, *F*(1, 31) = 13.05, *p* = 0.001. ${\eta^{2}}_{p}=$ 0.296. The LPP amplitude induced by high facial attractiveness (*M* = 2.04 μV, *SE* = 0.40) was significantly larger than that induced by low facial attractiveness (*M* = 0.45 μV, *SE* = 0.48). The main effect of employability was not observed, *F*(1, 31) = 2.94, *p* = 0.097, ${\eta^{2}}_{p}=$ 0.087.

**Figure 7:**
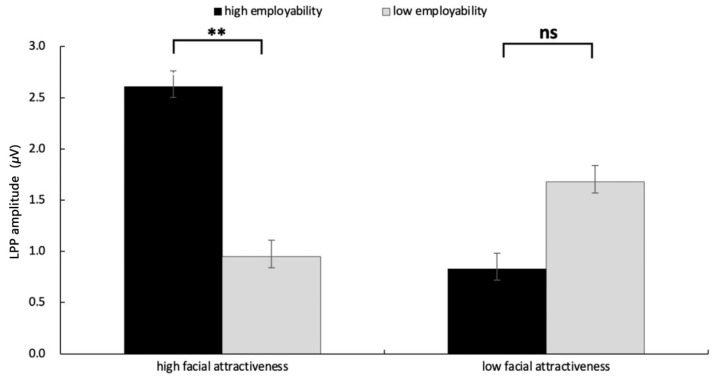
The results of LPP amplitude in each condition. Error bars represent standard errors (n.s.: nonsignificant, ** *p* < 0.01).

Additionally, the interaction between facial attractiveness and employability was significant, *F*(1, 31) = 4.25, *p* = 0.048, ${\eta^{2}}_{p}=$ 0.121. As illustrated in Figure 7, simple effects analysis revealed that, under the high facial attractiveness condition, LPP amplitude induced by high employability (*M* = 2.61 μV, *SE* = 0.62) was larger than that induced by low employability (*M* = 0.95 μV, *SE* = 0.54), *F*(1, 31) = 1.05, *p* = 0.013, ${\eta^{2}}_{p}=$ 0.033. However, under the low facial attractiveness condition, the difference between high and low employability was not significant (*p* = 0.35) (The ERP grand averaged waveforms and the topography are displayed in Figure 4 and Figure 5).

## 4. Discussion

The behavioral findings of this study revealed that participants rated job applicants with high facial attractiveness more favorably than those with low facial attractiveness, indicating that physical appearance plays a significant role in the resume evaluation process. This finding confirms the presence of appearance bias in hiring, highlighting how the physical appearance of job applicants can influence recruiter’s evaluation. Those with more favorable physical attributes leave a stronger impression, potentially increasing their competitiveness in the professional arena.

ERP results indicated that the study elicited the N170 effect related to employability during the early stages of perceptual and attentional processing. Under conditions of high facial attractiveness, lower employability resulted in a greater N170 amplitude compared to higher employability. This aligns with previous research (Tautvydaitė et al., 2022), suggesting that attention is quickly and automatically directed to stimuli perceived as potentially negative during the initial stages of facial processing. The main effect of employability on the P200 component was significant, with higher levels of employability eliciting larger P200 amplitudes regardless of facial attractiveness. This supports findings from previous research, which suggest that the P200 component is linked to the allocation of attentional resources (Boustani et al., 2021). Participants appear to allocate more attention to stimuli related to higher employability, reinforcing the importance of employability in early-stage evaluations.

In the later stages of processing, highly attractive faces elicited greater LPP amplitudes than their less attractive counterparts. This suggests that high facial attractiveness leads to stronger emotional experiences and increased emotional arousal. This finding is consistent with previous studies showing that appealing faces evoke larger LPP components during recognition (Marzi & Viggiano, 2010). The LPP component is often associated with the motivational significance of stimuli (Becker et al., 2024), with positive stimuli generally inducing greater LPP responses. The results of this study further support the idea that highly attractive faces evokes distinct emotional responses compared to less attractive ones, providing additional insights into how facial appearance influences emotional processing (Farkas et al., 2021).

Importantly, this study also revealed that facial attractiveness moderates the P200 and LPP effects evoked by employability. High employability enhanced these neural responses only when paired with high attractiveness, with no significant effects observed for less attractive faces. This may be due to the more positive perception of high attractiveness, which enhances emotional motivation toward high employability (Pell et al., 2022), leading to a stronger emotional response during the applicant evaluation process. Thus, the combination of attractiveness and employability may intensify individuals’ evaluation and categorization of emotional stimuli. From a cognitive neuroscience perspective, this study extends our understanding of how facial attractiveness influences applicant evaluations during the recruitment process, providing neurophysiological evidence for the effects of facial appearance in hiring decisions.

Practical implications: The results also carry practical implications for optimizing recruitment practices, particularly for managers seeking to mitigate biases and enhance fairness in organizational hiring processes.

To reduce the undue influence of facial attractiveness, managers can restructure the evaluation process to decouple visual cues from early-stage assessments; for instance, implementing a two-phase screening system where resumes are first reviewed in a “blind” format—with professional headshots, profile photos, or any visual identifiers temporarily redacted—can delay the introduction of facial attractiveness cues until later stages (e.g., interviews) when they may be more relevant (e.g., for client-facing roles).

Complementing such structural adjustments, leveraging Applicant Tracking Systems (ATS) by configuring them to prioritize objective employability metrics and filter out visual cues like headshots can further mitigate facial attractiveness bias in early-stage recruitment screenings. Modern ATS platforms can be programmed to focus exclusively on explicit qualifications—such as education, skills, and experience—during initial screenings, effectively sidelining visual data (e.g., photos) or non-essential personal details, which reduces the risk of subconscious attention to attractiveness that might otherwise overshadow competence signals. However, managers must pair this with rigorous algorithmic audits to prevent the reinforcement of other biases, as an ATS utilizing machine learning algorithms trained on historical hiring data may inherit and amplify existing human biases embedded in that data.

For job seekers, this study highlights the importance of not only meeting the qualifications for a position but also presenting themselves in a professional manner that aligns with the expectations of the organization. While qualifications should always be the primary focus, a well-rounded presentation—including how one communicates through their resume and during interviews—can play a significant role in the recruitment process.

## 5. Conclusions, Limitations and Future Directions

This study makes a contribution to understanding the interplay between facial attractiveness and employability in recruitment evaluations by integrating behavioral and neuroscientific approaches. By clarifying how these two factors jointly shape recruiters’ judgments—through both explicit ratings and distinct neural responses (N170, P200, LPP)—it bridges the gap between observable hiring biases and their subconscious neural origins. These findings not only advance theoretical insights into the cognitive and affective mechanisms underlying evaluative processes but also provide a neurophysiological foundation for developing evidence-based strategies to mitigate appearance-related biases, ultimately promoting fairer recruitment practices. By highlighting the dynamic interaction between implicit visual cues and explicit qualifications, this research enriches our understanding of hiring decision-making and offers actionable directions for reducing inequities in the workplace.

While this study advances our understanding of how facial attractiveness shapes neural processing during resume evaluation, it is important to acknowledge several limitations. First, the experimental design required participants to evaluate resumes with photographs, which may not reflect global hiring practices. In many Western countries (e.g., the United States, Canada, UK), including photos in resumes is generally discouraged due to anti-discrimination policies, meaning our findings may be most applicable to cultural contexts where photo-inclusive resumes are standard practice. This cultural variation highlights the need for cross-cultural replications to examine whether the observed neural effects persist in contexts where facial information is absent during initial screening.

Second, the current study did not include a neutral attractiveness condition. This decision was primarily driven by the consideration that neutral facial stimuli tend to exhibit greater perceptual variability among raters compared to high or low attractiveness levels, making them harder to operationalize reliably even when using validated face databases. However, the absence of a neutral condition may restrict the nuance of our findings. Without this reference point, it is challenging to precisely determine whether the observed neural effects are driven by the presence of high attractiveness, the absence of low attractiveness, or a more complex interplay between these two extremes. To address this gap, future research could incorporate neutral stimuli, which would help bridge our current neural findings with more detailed behavioral assessments of how attractiveness influences evaluative processes.

Third, while our participants had some hiring experience, they were still students rather than professional recruiters. Their judgments might differ from those of HR professionals with years of real-world hiring experience. Future studies should test these effects with actual hiring managers to confirm whether the findings hold in practice.

## Figures and Tables

**Table 1:** A Summary of behavioural data in four conditions.

Type	Applicant Evaluation Scores (1–5)			
	*M*	*SE*	*F*	*p*
High facial attractiveness	3.75	0.05	127.96	0.00
Low facial attractiveness	2.89	0.09		0.00
High employability	3.71	0.09	32.65	0.00
Low employability	2.93	0.09		0.00
interaction			4.93	0.00

Note. *SE* = standard error.

## Data Availability

The datasets generated during and/or analyzed during the current study are available from the author upon reasonable request.

## Note

1 Using G*Power, the acceptable values of ε fall within the range (0.333333, 1); thus, a median value of 0.67 was selected.

## Abbreviations

The following abbreviations are used in this manuscript:

ERPs	event-related potentials
EEG	Electroencephalography
LPP	Late positive potential
ANOVA	Analysis of variance

## Appendix A

**Table A1:** The enterprises as experiment materials.

Enterprises of China	Top 500	Non-Top 500
1	Youdao (网易有道)	Youwo (优渥农业)
2	China Telecom (中国电信)	Yanding (言鼎企业)
3	Jingdong (京东贸易)	Senlatianshi (硬制合金)
4	Alibaba (阿里巴巴)	Futuretech (博盈科技)
5	Gree (格力电器)	Niutron (牛创汽车)
6	Baidu (百度科技)	Bountif (佳裕农业)
7	Xiaomi (小米科技)	Wenkuang (文旷咨询)
8	Huawei (华为技术)	Fordwin (富源企业)
